# Colposcopic accuracy in diagnosing squamous intraepithelial lesions: a systematic review and meta-analysis of the International Federation of Cervical Pathology and Colposcopy 2011 terminology

**DOI:** 10.1186/s12885-023-10648-1

**Published:** 2023-02-23

**Authors:** Dongxu Qin, Anying Bai, Peng Xue, Samuel Seery, Jiaxu Wang, Maria Jose Gonzalez Mendez, Qing Li, Yu Jiang, Youlin Qiao

**Affiliations:** 1 School of Population Medicine and Public Health, Chinese Academy of Medical Sciences and Peking Union Medical College, Beijing, 100730 China; 2 Faculty of Health and Medicine, Division of Health Research, Lancaster University, Lancaster, LA1 4YW UK; 3 School of Public Health, Dalian Medical University, Dalian, 116044 Liaoning China; 4 Diagnosis and Treatment for Cervical Lesions Center, Shenzhen Maternity and Child Healthcare Hospital, Shenzhen, 518028 China

**Keywords:** Colposcopy, Diagnosis, Sensitivity, Specificity

## Abstract

**Background:**

Colposcopy is an important tool in diagnosing cervical cancer, and the International Federation of Cervical Pathology and Colposcopy (IFCPC) issued the latest version of the guidelines in 2011. This study aims to systematically assess the accuracy of colposcopy in predicting low-grade squamous intraepithelial lesions or worse (LSIL+) / high-grade squamous intraepithelial lesions or worse (HSIL+) under the 2011 IFCPC terminology.

**Methods:**

We performed a systematic review and meta-analysis, following the Preferred Reporting Items for Systematic Reviews and Meta-Analyses (PRISMA) guidelines. We searched for studies about the performance of colposcopy in diagnosing cervical intraepithelial neoplasia under the new IFCPC colposcopy terminology from PubMed, Embase, Web of Science and the Cochrane database. Data were independently extracted by two authors and an overall diagnostic performance index was calculated under two colposcopic thresholds.

**Results:**

Totally, fifteen articles with 22,764 participants in compliance with the criteria were included in meta-analysis. When colposcopy was used to detect LSIL+, the combined sensitivity and specificity were 0.92 (95% CI 0.88–0.95) and 0.51 (0.43–0.59), respectively. When colposcopy was used to detect HSIL+, the combined sensitivity and specificity were 0.68 (0.58–0.76) and 0.93 (0.88–0.96), respectively.

**Conclusion:**

In accordance with the 2011 IFCPC terminology, the accuracy of colposcopy has improved in terms of both sensitivity and specificity. Colposcopy is now more sensitive with LSIL+ taken as the cut-off value and is more specific to HSIL+. These findings suggest we are avoiding under- or overdiagnosis both of which impact on patients’ well-being.

**Supplementary Information:**

The online version contains supplementary material available at 10.1186/s12885-023-10648-1.

## Introduction

Cervical cancer ranks fourth among gynecological malignancies, with 600,000 new cases and 340,000 deaths worldwide in 2020 [1]. As a vital diagnostic tool and in the management of cervical cancer, colposcopy has become more commonly used around the world [2, 3]. However, colposcopy practice is not yet standardized and to promote a standardized approach, the International Federation of Cervical Pathology and Colposcopy (IFCPC) proposed a series of three terminologies, in 1975, 1990, and 2002. Then in 2011, the IFCPC nomenclature committee examined previous IFCPC terminologies and the existing knowledge before producing the first evidence-based terminology. The 2011 terminology is more comprehensive and was recommended to replace all previous terminologies [4–6]. Indeed, several studies conducted since 2011 have shown that this "new" terminology does improve colposcopic accuracy, if used correctly, and is clinically practicable [7, 8]. However, very few studies have assessed the 2011 IFCPC terminology [9] and no one has systematically reviewed the best available evidence for global communities.

Even though the relatively new IFCPC standard highlights the continuous development of colposcopic technologies and our understanding of colposcopic findings, the performance of these technologies in diagnosing squamous intraepithelial lesions varies substantially [9–11]. Fortunately, a number of technologies have emerged, such as dynamic spectral image (DSI) [12], smart phone [13], artificial intelligence [14, 15], and portable pocket colposcopy [16, 17]. These all help to provide more sophisticated analysis and therefore more appropriate diagnoses. However, it remains necessary to assess colposcopy performance against the gold standard biopsy. It is important to note that cervical biopsies take the form of a punch biopsy, endocervical curettage, or cone biopsy which are all invasive but are also only performed for colposcopy-based suspected cases. Of course, most physicians err on the side of caution, but colposcopy is a subjective process which requires skill and experience. Indeed, many clinicians and researchers alike have postulated that a comprehensive synthesis of the best available evidence would prove useful [18, 19].

In 2017, the American Society for Colposcopy and Cervical Pathology (ASCCP) has organized multiple working groups to draft colposcopy standards for the United States [20] After systematically reviewing 18 unique articles and synthesized knowledge, researchers recognized that there remains wide variation in both guidance and quality indicators. Crucially, the sample of studies was US centric and there have been a number of studies conducted around the world which may provide more generalizable insights. Therefore, it may be possible to yield more reliable findings if we assess colposcopic effectiveness from around the world, according to a set standard. Here, we systematically review (and meta-analyze) the evidence to assess the diagnostic performance of colposcopy-guided biopsies at different thresholds for the detection of histologically confirmed cervical intraepithelial neoplasia grade 2 or worse (CIN2+) according to the 2011 IFCPC terminology.

## Materials and methods

The study was developed and completed in compliance with the PRISMA checklist and the study protocol was registered in PROSPERO (CRD42021293845).

### Data source: search strategy and selection criteria

Relevant articles were identified using a set search strategy implemented in the following databases: PubMed, Embase, the Cochrane library and Web of Science from 1^st^ January, 2011, to 7^nd^ January, 2023. Searching began in May 2021 with two updated searches conducted in July 2022 and January 2023, before submitting for publication.

Search terms included "uterine cervical neoplasia", "squamous intraepithelial lesions", "colposcopy", "biopsy", "pathology", "sensitivity and specificity". In addition, we performed a reference list search to ensure all available evidence could be included or at least discussed. The detailed search strategy has been provided in the Supplementary materials.

Three thousand thirty-three articles were initially identified. Articles were included if they met three criteria: (1) The results of pathological examination were obtained using punch biopsy, cone biopsy, or hysterectomy specimens; (2) the article included raw data (not just aggregated data) in the form of a table comparing colposcopic impressions to results from colposcopy-guided biopsy, with results broken down according to either of the two independent histopathologic categories: the cervical intraepithelial lesions (CINs) system consisting of normal, CIN1, CIN2, CIN3 and cancer, or the LAST system including normal, low squamous intraepithelial lesions (LSIL), high squamous intraepithelial lesions (HSIL) and cancer; and (3) all results had been determined in accordance with the 2011 IFCPC terminology.

The following studies were excluded: duplicate publications; reviews; editorials; non-human samples; and no studies of colposcopy for detecting CINs. Duplicates were manually removed using Endnote software (version X9). Two authors independently screened the titles and abstracts according to these eligibility criteria, and relevant articles for full text were downloaded and reviewed. Any disagreement was resolved through discussion with a third author.

### Data extraction and risk-of-bias assessment

Two cut-off values were set in this study. First, if colposcopy results suggested normal or benign, the patients were categorized as <LSIL, and if the results were CIN1, CIN2, CIN3, LSIL, HSIL, or cancer, we categorized patients as LSIL+. Second, if the colposcopy results of patients were considered normal, benign, CIN1 or LSIL, patients were considered <HSIL, and if the results were CIN2, CIN3, HSIL, or cancer, we considered the patients as HSIL+. To unify the criteria, we combined CIN1, CIN2, CIN3, LSIL, HSIL, and cancer into LSIL or worse (LSIL+), and combined CIN2, CIN3, HSIL and cancer into HSIL or worse (HSIL+). The predictive value of colposcopy in diagnosing CINs was based on its accuracy for detecting HSIL+ (confirmed by histopathology).

Data extraction from eligible articles was performed by one author, then two independent authors compiled the data into a standardized table, while the other author cross-checked the extracted information. Disagreements were resolved by a third author.

Extracted information included publication year, number of patients, time of recruitment, sensitivity, specificity, positive predictive value (PPV), and negative predictive value (NPV). If these data were not provided directly, we back calculated the required values using the reported data.

Study quality was assessed using the Quality Assessment of Diagnostic Accuracy Studies (QUADAS-2) [21], which consists of four domains: 1) patient selection; 2) index test; 3) reference standard; and 4) flow and timing. The first three domains can also be used to assess applicability. Two authors independently assessed quality of the included reports and conflicts were resolved through discussion. Details of quality assessment can be seen in Figure S1.

### Statistical analysis

Sensitivity and specificity estimates, according to different cut-off values for colposcopic diagnosis, were calculated by cross-tabulation. Forest plots were generated for each test, with the corresponding 95% confidence intervals (95% CIs). Pooled estimates for test accuracy are presented graphically with Summary Receiver-Operating Characteristic (SROC) curves. Summary points, area under the receiver operating curve (AUC) with 95% CIs, and prediction contours are also described. The risk of publication bias was assessed statistically using a funnel plot, and egger’s regression test. STATA (version 16.0) and RevMan (version 5.4) were used for all data analyses.

## Results

### Study characteristics

**Fig. 1:**
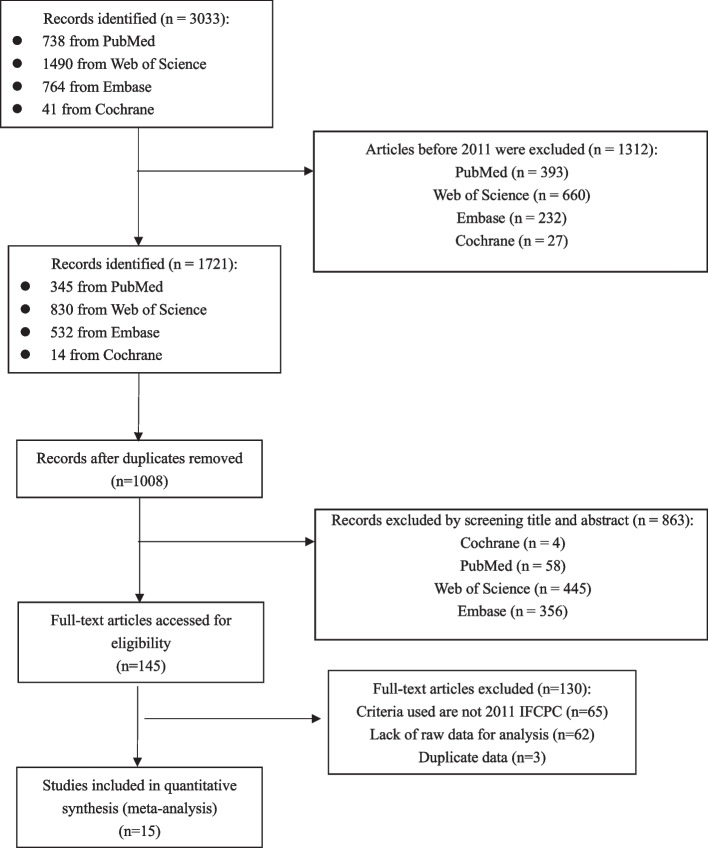
Selection and inclusion process of included studies

A total of 3,033 studies were identified, of which 1312 before 2011 were excluded. Among the remaining 1721 records, 1008 were left behind and 713 were excluded because of duplication, and 863 were excluded at the initial screening of abstracts. The remaining 145 full-text articles were assessed, and a further 130 studies were excluded for not fulfilling our predetermined inclusion criteria (Fig. 1). Finally, a total of 15 [7–9, 22–32] articles with 22,764 participants in compliance with the criteria were included in the study.

Fifteen studies met the data requirements for meta-analysis in the <LSIL category compared with LSIL+, and <HSIL compared to HSIL+. Sensitivity and specificity were calculated using these two thresholds, independently. Overall likelihood, SROC curves, sensitivity, specificity, and AUCs were calculated. Two SROC curves were estimated using meta-analysis from all ten independent studies’ sensitivity and specificity, which reflect the overall performance of colposcopy as a diagnostic tool.

Additionally, calculated the AUC results could be used to compare differences. Table 1 summarizes the demographic characteristics of the participants in the included articles. Tables 2 and 3 report the sensitivity, specificity, and their 95% CI, as well as true positive, true negative, false positive, and false negative using two thresholds separately from the 14 included publications, which are all important indicators of diagnostic accuracy.

**Table 1:** Participant demographics for 15 included studies

	**Study design**	**Country**	**Inclusion criteria**	**Exclusion criteria**	**Type of colposcope**	**Sample size**	**Time of recruitment**	**Number and type of biopsies**	**Referral cytology/HPV testing**	**The level of colposcopists**	**Mean age (SD; range)**	**Main Conclusion**
Ghosh et al., 2014 [24]	Retrospective	India	Intact uterus; without a history of precancer/cancer; nonpregnant	NR	NR	2466	2011–2013	≥ 1; abnormal areas	HC2	≥ 10 years	NR (NR; 30–60)	Colposcopy performed well in the overall detection of cervical neoplasias, though its capability for accurate categorisation of degree of abnormality was poor
Spinillo et al., 2014 [28]	Retrospective	Italy	NR	Pregnancy, treatment, hysterectomy;	NR	2526	2009–2012	2–4; abnormal areas and random biopsies	Thinprep cytology; HPV	NR	37 (29–45; NR)	Multiple infection or HPV16 positivity did not affect colposcopic accuracy in the diagnosis of CIN3+ lesions. The sensitivity of colposcopy was poor among subjects who were uninfected or infected by low-risk HPV genotypes
Zhao et al., 2015 [30]	Retrospective	China	No history of hysterectomy, pregnancy, pelvic radiotherapy, screening	NR	NR	1997	1999	2–4; abnormal areas and random biopsies	Thinprep cytology; HC2	NR	39.6 (3.2;35–45)	4-quadrant biopsy can detect more HSIL+ lesions and is more accurate than suspicious lesion biopsy alone
Coronado et al., 2016 [22]	Retrospective	Spain	Aged ≥ 18 years	NR	Digital colposcopy	443	2012–2014	2; abnormal areas	Pap smear; HPV	Accredited as experts by the SSCPC	36.0 (10.9; NR)	Combining conventional colposcopy with DSI mapping improves the capability to detect cervical lesions
Li et al., 2017 [8]	Retrospective	China	NR	Had a history of hysterectomy or treatment; incomplete data	Digital colposcopy	525	2014–2015	≥ 1;abnormal areas and random biopsies	HC2 or Cobas HPV	Received training by 2011 IFCPC	40.13 (10.23; NR)	The 2011 IFCPC nomenclature improves colposcopic accuracy in trained colposcopists, like speaking the same language. However, the reproducibility of TZ and the predictive value of a few signs remain to be discussed
Fan et al., 2018 [9]	Retrospective	China	No treatment history hysterectomy; no clinically suspected immunosuppression	NR	Digital colposcopy	2262	2012–2016	≥ 1;abnormal areas and random biopsies	Cytology; HR-HPV	≥ 5 years	41.3 (11.6; NR)	The 2011 IFCPC terminology can improve the diagnostic accuracy for all lesion severities. The categorization of major changes and minor changes is appropriate. However, colposcopic diagnosis remains unsatisfactory
Liu et al., 2018 [31]	Retrospective	China	Complete case data	Patients with a history of pathology or surgery and pelvic radiotherapy	NR	256	2014–2016	≥ 1; abnormal areas and random biopsies	Pap smear; NR	Senior	47 (NR;23–80)	The type of transformation zone is positively correlated with the age, and it can help to choose biopsy and therapeutic manner. The diagnostic accuracies of HSIL and early stage of cervical cancer by multi-point biopsy of colposcopy and/or ECC are high
Ruan et al., 2020 [27]	Retrospective	China	NR	Unclear image or the patients lacked the image; no HPV, cytology or histopathology	Digital colposcopy	1828	2016–2019	NR	Cytology; HPV	≥ 20 years	37 (NR; 17–81)	The data and findings herein provide the resource for evaluating the diagnostic value of colposcopy, and suggested that the accuracy of colposcopy is required to be further improved
Del Pino et al., 2021 [23]	Prospective	Spain	NR	Previous treatment; pregnancy;	Digital colposcopy	302	2014–2015	2–4; abnormal areas and random biopsies	ThinPrep cytology; Cobas HPV	accredited by the SSCPC	37.6 (10.3; NR)	Colposcopy impression provides essential information to identify women at risk of HSIL/CIN3
Li et al., 2021 [25]	Retrospective	China	NR	No screening results, had uterectom or CIN; pregnant or with incomplete data	NR	495	2017–2019	≥ 1;abnormal areas and random biopsies	ThinPrep cytology; HC2, genotype	NR	40 (NR; 21–71)	Colposcopy is an excellent tool to estimate cervical high-grade lesion but is imprecise. Many factors can bias the diagnosis of colposcopy, especially the known results of cervical cytology and HPV
Liu et al., 2021 [26]	Retrospective	China	NR	Pregnancy; previous lesions or surgery, hysterectomy; incomplete data	Digital colposcopy	987	2015–2019	≥ 1;abnormal areas	Cytology	NR	41.94 (12.45; NR)	The diagnostic value of IFCPC and R-way is better than Reid. There is good agreement between R-way colposcopy evaluation and histopathology
Zhang et al., 2022 [33]	Retrospective	China	NR	Had hysterectomy or pelvic radiation; no histopathology	Digital colposcopy	1838	2013–2018	2–4; abnormal areas and random biopsies	Cytology; HR-HPV	5–7 years	41.7 (10.6; NR)	Positive p16(INK4a) immunostaining is very strongly consistent with an H&E diagnosis of CIN2+, and it can be used as an objective detection index for HSIL + diagnoses of HPV-negative patients with CIN2+
Maffini et al., 2022 [34]	Retrospective	Brazil	Aged ≥ 21 years; no treatment; no history of hysterectomy	No colposcopic records; inadequate cytology review	NR	102	2009–2016	Biopsy the worst area	Pap smear; NR	30 years	36 (NR; 21–84)	Colposcopy performed by an experienced examiner can accurately differentiate patients with CIN1 or less from patients with CIN2 or worse. Diagnosis of CIN2 or worse was more frequent in patients with a previous history of cervical dysplasia and pre-menopausal patients. The degree of acetowhite changes was the best colposcopic feature to predict CIN2 or worse
Wei et al., 2022 [29]	Retrospective	China	NR	Had treatment or hysterectomy; had no histologic report	Digital colposcopy	2417	2018–2021	Abnormal areas and random biopsies	NR	Senior and junior	NR	It appears possible to supplement colposcopic examinations with screening results to improve HSIL+ detection, especially for women with TZ3 lesions. It may also be possible to improve junior colposcopists' diagnoses although, further psychological research is necessary
Stuebs et al., 2022 [32]	Retrospective	Erlangen	Had a biopsy or underwent excisional surgical treatment—LLETZ, loop electrosurgical excision procedure with laser coagulation of the periphery or laser conization	Without a biopsy being taken during the colposcopic examination	Digital colposcopy	4778	2015–2022	Biopsy the worst area	Pap smear; NR	Experienced and highly qualified	36.8(10.8, NR)	Colposcopy is an important, feasible, and effective method. Careful work-up needs to be performed for women with TZ3 who are over 35 years old, as they are at the highest risk of being misdiagnosed. The highest concordance for detecting HSIL + was seen for colposcopists with > 10 years’ experience

*Abbreviations:* *NR* Not reported, *SD* Standard deviation, *CIN* Cervical intraepithelial neoplasia, *HC2* Hybrid capture 2 tests, *SSCPS* Spanish society of cervical pathology and colposcopy, *CSCCP* Chinese society for colposcopy and cervical pathology

**Table 2:** Effectiveness of colposcopy in distinguishing <LSIL from LSIL+

Study	TP	FP	FN	TN	Total	Sensitivity (95% CI)	Specificity (95% CI)
Ghosh et al., 2014 [24]	196	1705	32	533	2466	0.86 (0.81–0.90)	0.24 (0.22–0.26)
Spinillo et al., 2014 [28]	432	1013	103	978	2526	0.81 (0.77–0.84)	0.49 (0.47–0.51)
Zhao et al., 2015 [30]	70	449	16	1462	1997	0.81 (0.72–0.89)	0.77 (0.75–0.78)
Coronado et al., 2016 [22]	38	123	3	279	443	0.93 (0.80–0.98)	0.69 (0.65–0.74)
Li et al., 2017 [8]	90	195	9	231	525	0.91 (0.83–0.96)	0.54 (0.49–0.59)
Fan et al., 2018 [9]	612	901	19	730	2262	0.97 (0.95–0.98)	0.45 (0.42–0.47)
Liu et al., 2018 [31]	30	112	0	114	256	1.00 (0.88–1.00)	0.50 (0.44–0.57)
Ruan et al., 2020 [27]	430	538	71	789	1828	0.86 (0.82–0.89)	0.59 (0.57–0.62)
Del Pino et al., 2021 [23]	98	88	25	91	302	0.80 (0.71–0.86)	0.51 (0.43–0.58)
Li et al., 2021 [25]	289	114	9	83	495	0.97 (0.94–0.99)	0.42 (0.35–0.49)
Liu et al., 2021 [26]	160	328	10	489	987	0.94 (0.89–0.97)	0.60 (0.56–0.63)
Zhang et al., 2022 [33]	603	878	12	345	1838	0.98 (0.97–0.99)	0.28 (0.26–0.31)
Maffini et al., 2022 [34]	69	6	9	18	102	0.88 (0.79–0.95)	0.75 (0.53–0.90)
Wei et al., 2022 [29]	1028	618	55	716	2417	0.95 (0.93–0.96)	0.54 (0.51–0.56)
Stuebs et al., 2022 [32]	2397	1373	281	727	4778	0.90 (0.88–0.91)	0.35 (0.33–0.37)

*Abbreviations:* *LSIL* Low squamous intraepithelial lesions, *HSIL* High squamous intraepithelial lesions, *TP* True positive, *TN* True negative, *FP* False positive, *FN* False negative

**Table 3:** Effectiveness of colposcopy in distinguishing <HSIL from HSIL+

Study	TP	FP	FN	TN	Total	Sensitivity (95% CI)	Specificity (95% CI)
Ghosh et al., 2014 [24]	120	194	108	2044	2466	0.53 (0.46–0.59)	0.91 (0.90–0.92)
Spinillo et al., 2014 [28]	195	91	340	1900	2526	0.36 (0.32–0.41)	0.95 (0.94–0.96)
Zhao et al., 2015 [31]	36	20	50	1891	1997	0.42 (0.31–0.53)	0.99 (0.98–0.99)
Coronado et al., 2016 [22]	33	40	8	362	443	0.80 (0.65–0.91)	0.90 (0.87–0.93)
Li et al., 2017 [8]	63	17	36	409	525	0.64 (0.53–0.73)	0.96 (0.94–0.98)
Fan et al., 2018 [9]	452	33	179	1598	2262	0.72 (0.68–0.75)	0.98 (0.97–0.99)
Liu et al., 2018 [31]	30	42	0	184	256	1.00 (0.88–1.00)	0.81 (0.76–0.86)
Ruan et al., 2020 [27]	282	82	219	1245	1828	0.56 (0.52–0.61)	0.94 (0.92–0.95)
Del Pino et al., 2021 [23]	69	29	54	150	302	0.56 (0.47–0.65)	0.84 (0.78–0.89)
Li et al., 2021 [25]	163	12	135	185	495	0.55 (0.49–0.60)	0.94 (0.90–0.97)
Liu et al., 2021 [26]	127	46	43	771	987	0.75 (0.67–0.81)	0.94 (0.93–0.96)
Zhang et al., 2022 [33]	485	89	130	1134	1838	0.79 (0.75–0.82)	0.93 (0.91–0.94)
Maffini et al., 2022 [34]	64	0	14	24	102	0.82 (0.72–0.90)	1.00 (0.86–1.00)
Wei et al., 2022 [29]	760	332	323	1002	2417	0.70 (0.67–0.73)	0.75 (0.73–0.77)
Stuebs et al., 2022 [32]	2082	644	596	1456	4778	0.78 (0.76–0.79)	0.69 (0.67–0.71)

*Abbreviations:* *LSIL* Low squamous intraepithelial lesions, *HSIL* High squamous intraepithelial lesions, *TP* True positive, *TN* True negative, *FP* False positive, *FN* False negative

**Fig. 2:**
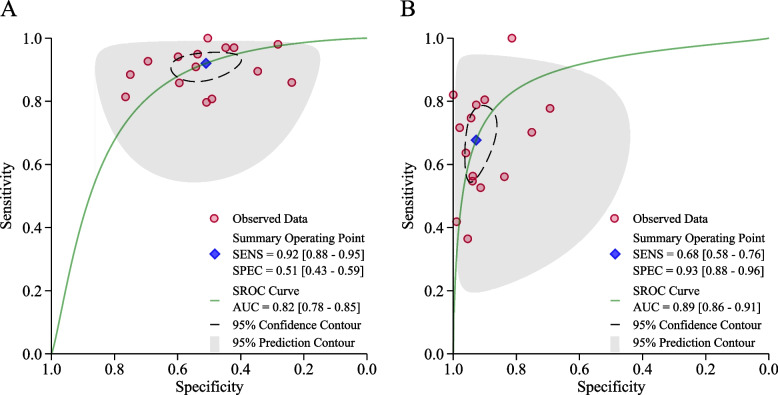
Sensitivity and specificity reported for diagnostic colposcopic impression in 14 studies (each study represented by a point in the figure) relative to the gold standard of biopsy for distinguishing **A** <LSIL from LSIL+; **B** <HSIL from HSIL+. The solid line in the graph shows the receiver operating characteristic curve determined from regression analysis

**Fig. 3:**
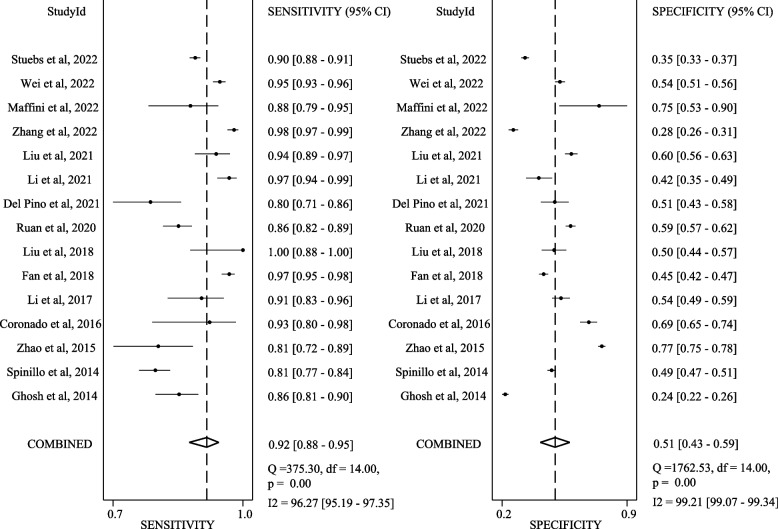
Sensitivity and specificity reported for distinguishing  <LSIL from LSIL+

**Fig. 4:**
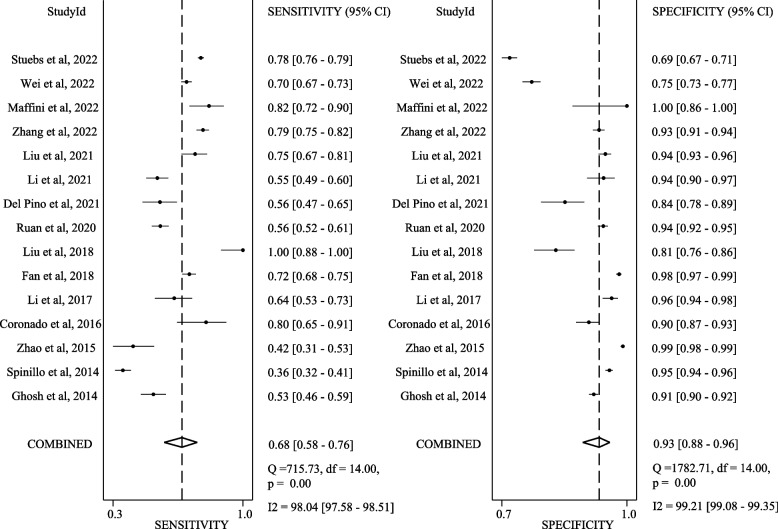
Sensitivity and specificity reported for distinguishing <HSIL from HSIL+

Figures 2 present SROCs with prediction and confidence contours and AUC with 95% CI. Figures 3 and 4 present the sensitivity and specificity data with 95% CI from each study at the different cut-offs reported. Figure S2 present the Deek’s funnel-based asymmetry test. The p-value for Deek’ s asymmetry is 0.06, and the egger’s (*P* = 0.064) regression tests likewise were statistically insignificant, indicating no publication bias.

### Pooled performance of colposcopy under two thresholds

When testing colposcopy with a cut-off of LSIL+, the pooled sensitivity is 0.92 (95% CI 0.88–0.95), the pooled specificity is 0.51 (95% CI 0.43–0.59), summary SROC analysis confirmed the ability of colposcopy in distinguishing <LSIL from LSIL+, with a mean (SE) AUC of 0.82 (95% CI 0.78–0.85). I^2^ of sensitivity is 96.27%, of specificity is 99.21%. When testing colposcopy with a cut-off of HSIL+, the pooled sensitivity is 0.68 (95% CI 0.58–0.76), the pooled specificity is 0.93 (95% CI 0.88–0.96), summary SROC analysis confirmed the ability of colposcopy in distinguishing <HSIL from HSIL+, with a mean (SE) AUC of 0.89 (95% CI 0.86–0.91). I^2^ of sensitivity is 98.04%, of specificity is 99.21%.

## Discussion

This study aimed to assess the accuracy of colposcopy based on histopathologic findings according to the 2011 IFCPC terminology, which might be helpful to generate generalizable findings by synthesizing evidence from studies using the same terminology around the world. We systematically reviewed (and meta-analyzed) evidence to assess the performance of colposcopy at different thresholds. Our results suggest that the sensitivity of colposcopy diagnosis is high (0.92), although with relatively low specificity (0.51) when LSIL is adopted as the cut-off value. Conversely, using HSIL+ as the cut-off value appears to lower the sensitivity (0.68) and raise specificity (0.93). AUC analysis also indicates that there is a higher level of overall accuracy using HSIL+ as the threshold (0.89 vs 0.82). Quality assessment suggests that the included studies are high quality, and there was no apparent publication bias, despite having included only nine studies.

The wide range of values for both sensitivity and specificity found in each of the 15 included studies were very similar to the ranges reported in reviews by Brown et al. [35] and Underwood et al. [36]. Findings around overall sensitivity (0.68) and specificity (0.93) when using HSIL+ as the cut-off value, were also equivalent to previous studies [37–39]. The sensitivity of colposcopy for HSIL+ from 49 to 61% [40], and specificity varied from 79 to 96.5%. Given that our results are within these ranges, approximately 40% of CIN2+ cases are missed at initial colposcopy when using this threshold. This is far too high and requires our immediate attention because late diagnosis limits the number and efficacy of treatment options. However, previous research also showed that over one-third of all CIN2+ cases would progress into cervical cancer over a period of between 10 and 15 years [41], while the change of missed lower grade lesions in progressing into invasive disease was little, which justified advocating HSIL+ as the more clinically meaningful cut-off value regardless of the lower sensitivity.

In this study, the diagnostic performance for detecting CIN2+ was calculated for colposcopic impressions using both cut-off values, i.e., LSIL+ and HSIL+. When the cut-off value of colposcopic impressions was LSIL+, the lower specificity became normal, as some of the patients with low-grade colposcopic diagnosis may not have pathologic CIN2+. Biopsy of all suspected lesions, i.e., LSIL+, appears to result in the highest sensitivity for detecting CIN2+, which is the main biopsy strategy used in low- and middle-income countries (LMICs). For example, this approach is commonly used in China, because it is difficult to accurately grade LSIL and HSIL lesions. Evidence from this study therefore recommends a balanced cut-off value for low-grade (or worse) to reduce the number of missed CIN2+ cases, even though specificity drops.

It should be noted that verification bias is a particular problem in studies of colposcopy. This is because of the process involved and the economic pressures health systems face. Biopsies are only performed in suspected cases and as a result, biopsies often become the process of verification rather than investigation. If a biopsy is not taken after colposcopy results are negative, then the sensitivity might be 100% which re-affirms clinical decisions. Additionally, spectrum bias might occur due to diversity in disease prevalence. Even though the incidence of a disease would not change sensitivity and specificity calculations within the test population, this can affect a sample consisting of disease negative participants [35]. Again, this has a knock-on effect and creates ambiguity from screening to diagnosis, which causes unnecessary anguish, raises the price of healthcare, and ultimately costs lives.

While colposcopy is increasingly common in cervical cancer screening, it is, as we have mentioned, a subjective procedure. A number of researchers have found correlations between colposcopy and histopathology are all too often misleading and generally unsatisfactory [42]. For example, Ruan et al. [27] found that colposcopy often underestimates the occurrence of squamous intraepithelial lesions when using biopsy as the pathologic gold standard. By contrast, Tatiyachonwiphut et al. [38] found that colposcopic diagnoses more often overestimate the incidence of cervical pathologies. These discrepancies might be due to the use of different colposcopic thresholds and methods; however, evidence from this meta-analysis suggests that LSIL may be selected as the cut-off value for directing biopsy in areas with underdeveloped colposcopy, while HSIL+ can be selected as a cut-off value to avoid unnecessary diagnosis. Moreover, digital colposcopy has potential values in providing accurate and objective measurements of a number of cervical features. Some studies reported high sensitivities and specificities concerning digital colposcopy compared with traditional colposcopy [43], while its wide application may be limited by the relatively high purchase and maintenance costs, an important factor especially in lower resource areas.

One of the reasons for conducting this systematic review was to provide support for global communities. The distribution of medical resources is disproportionate, which means there are fewer senior colposcopists in LMICs which directly affects women’s health and well-being [40]. Therefore, to meet the challenge of elimination of cervical cancer in LMICs, studies exploring feasible methods to improve the diagnostic performance of colposcopy are needed. Bekkers et al. [44] has found that junior colposcopists were significantly more likely to require biopsy compared to more senior colposcopists. Due to a lack of experience, junior colposcopists tend to order biopsies when in doubt. Conversely, increased confidence in colposcopic assessment displayed by more senior colposcopists might result in higher positive predictive values, but this is often at the expense of lower sensitivity. Therefore, junior colposcopists might not be able to identify HSIL+ cases accurately based on colposcopic images, and therefore refer to perform more biopsies for final confirmation.

In these circumstances, LSIL+ should be used as the cut-off value to reduce the number of false negatives. Since colposcopic biopsy is the gold standard for cervical cancer, it is important to improve the accuracy of colposcopy to improve identification processes. Specific training is compulsory before practitioners can be certified as colposcopists in some countries [44]. In LMICs, the quality of colposcopists could be effectively improved by increasing the amount and standard of training and by giving more professional guidance to uncertified colposcopists. However, enhancing quality control and advocating novel training methods, such as widely applicable teaching equipment [45] and training software [46], could also help to enhance colposcopists’ skills. This study suggests that skills vary substantially and that the application of the IFCPC terminology may also vary, which requires further research.

This systematic review identified gaps in our knowledge and some methodological issues that should be considered in future studies of cervical screening. Standardizing the evaluation of colposcopy based on the 2011 IFCPC, it could not only help to provide a reference for colposcopists, but also highlights emerging techniques for assessment [47]. Even though there are many alternative options for cervical cancer screening, Sawaya et al. [48] suggests that studies directly assessing the accuracy of screening tests or comparing between test results and colposcopy are inconclusive. Under limited resource settings [49], objective methods, such as molecular HPV testing, may be more appropriate. Until now, some of these methods have not been universally accepted due to concerns about health resource conditions in certain areas such as sub-Saharan Africa [50]. Therefore, colposcopy still remains fundamentally important in high-income countries and increasingly useful in LMICs.

### Strengths and limitations

While this is the first systematic review with meta-analysis of the diagnostic value of colposcopy based on the latest version of IFCPC guidelines, there were some limitations that could not be avoided. First, because of the strict screening criteria used in this study, the number of studies included in this analysis was relatively small. This clearly reduces the generalizability of the findings and means that recommendations can only be tentative. We attempted to quantify the diagnostic performance of colposcopy under two cut-off values which underpin its utility in clinical practice. However, biases in the design of the included studies also made the interpretation of our findings less than certain. It also emerged that medical variability is apparent and that we were unable to plan or extract information regarding experiential and skillset differences. This is certainly something that requires further attention, and perhaps would be best looked at by health economists and medical educationalists who could consider understanding the impact of educational strategies on patients.

## Conclusions

This meta-analysis confirmed the diagnostic value of colposcopy, as an effective tool for diagnosing cervical lesions. High sensitivity was observed with the LSIL+ cut-off, while high specificity was observed with a HSIL+ cut-off for squamous intraepithelial lesions. This might be used to provide guidance for future clinical practice and colposcopic research. Although, further research into the impact of colposcopy-based educational strategies is required.

## Supplementary Information

**Additional file 1:** Search strategies of identification of studies. **Figure S1.** Details of quality assessment by the QUADAS-2 tool. **Figure S2.** Deeks’ Funnel Plot Asymmety.

## Data Availability

The data generated in the present study may be requested from the corresponding authors.
