# Supplementary material for: Colposcopic accuracy in diagnosing squamous intraepithelial lesions: a systematic review and meta-analysis of the International Federation of Cervical Pathology and Colposcopy 2011 terminology
Source: BMC Cancer. 2023 Feb 23;23:187. doi: 10.1186/s12885-023-10648-1 (PMC9951444; doi:10.1186/s12885-023-10648-1)
Supplement: Supplementary file 1 — Additional file 1: Search strategies of identification of studies. Figure S1. Details of quality assessment by the QUADAS-2 tool. Figure S2. Deeks’ Funnel Plot Asymmety. [file 12885_2023_10648_MOESM1_ESM.pdf]

# Supplementary Materials

## 1．Search strategies of identification of studies

**Cochrane-41**

#1 MeSH descriptor: [Uterine Cervical Neoplasms] explode all trees

#2 (Uterine Cervical Neoplasms):ti,ab,kw OR (Cancer of the Uterine Cervix):ti,ab,kw OR (Cervix Cancer):ti,ab,kw OR (Cancer of Cervix):ti,ab,kw AND (Cancer of the Cervix):ti,ab,kw

#3 MeSH descriptor: [Squamous Intraepithelial Lesions] explode all trees

#4 (Squamous Intraepithelial Lesion):ti,ab,kw OR (Low Grade Squamous Intraepithelial Lesion):ti,ab,kw OR (High Grade Squamous Intraepithelial Lesion):ti,ab,kw OR (LSIL):ti,ab,kw OR (HSIL):ti,ab,kw

#5 #1 OR #2 OR #3 OR #4

#6 MeSH descriptor: [Colposcopy] explode all trees

#7 (Colposcopic Surgery):ti,ab,kw OR (Colposcopic Surgical Procedure):ti,ab,kw OR (Colposcopic Surgeries):ti,ab,kw OR (Colposcopies):ti,ab,kw

#8 #6 OR #7

#9 MeSH descriptor: [Diagnosis] explode all trees

#10 (Diagnose):ti,ab,kw OR (Diagnoses and Examinations):ti,ab,kw OR (Examinations and Diagnoses):ti,ab,kw OR (Antemortem Diagnosis):ti,ab,kw OR (Postmortem Diagnosis):ti,ab,kw

#11 #9 OR #10

#12 MeSH descriptor: [Pathology] explode all trees

#13 (Pathology):ti,ab,kw

#14 #12 OR #13

#15 MeSH descriptor: [Sensitivity and Specificity] explode all trees

#16 (Sensitivity):ti,ab,kw OR (Specificity):ti,ab,kw OR (Specificity and Sensitivity):ti,ab,kw OR (Sensitivity and Specificity):ti,ab,kw

#17 #15 OR #16

#18 #5 AND #8 AND #11 AND #14 AND #17

**Embase-764**

#42 #17 AND #20 AND #30 AND #36 AND #41

#41 #37 OR #38 OR #39 OR #40

#40 'sensitivity':ab,ti

#39 'specificity':ab,ti

#38 'specificity and sensitivity':ab,ti

#37 'sensitivity and specificity'/exp

#36 #31 OR #32 OR #33 OR #34 OR #35

#35 'pathology institute':ab,ti

#34 'pathologic institute':ab,ti

#33 'pathobiology':ab,ti

#32 'clinical pathology':ab,ti

#31 'pathology'/exp

#30 #21 OR #22 OR #23 OR #24 OR #25 OR #26 OR #27 OR #28 OR #29

#29 'physical diagnosis':ab,ti

#28 'medical diagnosis':ab,ti

#27 'disease diagnosis':ab,ti

#26 'diagnostics':ab,ti

#25 'diagnostic tool':ab,ti

#24 'diagnostic sign':ab,ti

#23 'diagnostic screening programs':ab,ti

#22 'diagnostic screening':ab,ti

#21 'diagnosis'/exp

#20 #18 OR #19

#19 'kolposcopy':ab,ti

#18 'colposcopy'/exp

#17 #1 OR #2 OR #3 OR #4 OR #5 OR #6 OR #7 OR #8 OR #9 OR #10 OR #11 OR #12 OR #13 OR #14 OR #15 OR #16

#16 'uterine cervix tumour':ab,ti

#15 'uterine cervix neoplasm':ab,ti

#14 'uterine cervical neoplasms':ab,ti

#13 'cervix tumour':ab,ti

#12 'cervix tumor':ab,ti

#11 'cervix neoplasms':ab,ti

#10 'cervix neoplasia':ab,ti

#9 'squamous intraepithelial lesion of the cervix'/exp

#8 'uterine cervix tumour':ab,ti

#7 'uterine cervix neoplasm':ab,ti

#6 'uterine cervical neoplasms':ab,ti

#5 'cervix tumour':ab,ti

#4 'cervix tumor':ab,ti

#3 'cervix neoplasms':ab,ti

#2 'cervix neoplasia':ab,ti

#1 'uterine cervix tumor'/exp

**PubMed-738**

((Uterine Cervical Neoplasms[MeSH Terms]) **OR** (Cervical Neoplasm, Uterine[Title/Abstract]) **OR** (Neoplasm, Uterine Cervical[Title/Abstract]) **OR** (Uterine Cervical Neoplasm[Title/Abstract]) **OR** (Neoplasms, Cervical[Title/Abstract]) **OR** (Cervical Neoplasms[Title/Abstract]) **OR** (Cervical Neoplasm[Title/Abstract]) **OR** (Neoplasms, Cervix[Title/Abstract]) **OR** (Cervix Neoplasm[Title/Abstract]) **OR** (Neoplasm, Cervix[Title/Abstract]) **OR** (Cervix Neoplasms[Title/Abstract]) **OR** (Cancer of the Uterine Cervix[Title/Abstract]) **OR** (Cancer of the Cervix[Title/Abstract]) **OR** (Cervical Cancer[Title/Abstract]) **OR** (Cancer, Cervical[Title/Abstract]) **OR** (Cervical Cancers[Title/Abstract]) **OR** (Uterine Cervical Cancer[Title/Abstract]) **OR** (Cancer, Uterine Cervical[Title/Abstract]) **OR** (Cervical Cancer, Uterine[Title/Abstract]) **OR** (Uterine Cervical Cancers[Title/Abstract]) **OR** (Cancer of Cervix[Title/Abstract]) **OR** (Cervix Cancer[Title/Abstract]) **OR** (Cancer, Cervix[Title/Abstract]) **OR** (Squamous Intraepithelial Lesions[MeSH Terms]) **OR** (Intraepithelial Lesion, Squamous[Title/Abstract]) **OR** (Lesions, Squamous Intraepithelial[Title/Abstract]) **OR** (Squamous Intraepithelial

Lesion[Title/Abstract]) **OR** (LSIL, Atypical Squamous Cells Cannot Exclude HSIL[Title/Abstract]) **OR** (LSIL ASC-H[Title/Abstract]) **OR** (High-Grade Squamous Intraepithelial Lesions[Title/Abstract]) **OR** (High Grade Squamous Intraepithelial Lesions[Title/Abstract]) **OR** (HSIL, High-Grade Squamous Intraepithelial Lesions[Title/Abstract]) **OR** (HSIL, High Grade Squamous Intraepithelial Lesions[Title/Abstract]) **OR** (HSIL, High Grade Squamous Intraepithelial Lesion[Title/Abstract]) **OR** (High-Grade Squamous Intraepithelial Lesion[Title/Abstract]) **OR** (High Grade Squamous Intraepithelial Lesion[Title/Abstract]) **OR** (Low-Grade Squamous Intraepithelial Lesions[Title/Abstract]) **OR** (Low Grade Squamous Intraepithelial Lesions[Title/Abstract]) **OR** (Low-Grade Squamous Intraepithelial Lesion[Title/Abstract]) **OR** (Low Grade Squamous Intraepithelial Lesion[Title/Abstract]) **OR** (LSIL, Low-Grade Squamous Intraepithelial Lesions[Title/Abstract]) **OR** (LSIL, Low Grade Squamous Intraepithelial Lesions[Title/Abstract]) **OR** (LSIL, Low-Grade Squamous Intraepithelial Lesion[Title/Abstract]) **OR** (LSIL, Low Grade Squamous Intraepithelial Lesion[Title/Abstract])) **AND** ((Colposcopy[MeSH Terms]) **OR** (Colposcopies[Title/Abstract]) **OR** (Colposcopic Surgical Procedures[Title/Abstract]) **OR** (Colposcopic Surgical Procedure[Title/Abstract]) **OR** (Procedure, Colposcopic Surgical[Title/Abstract]) **OR** (Procedures, Colposcopic Surgical[Title/Abstract]) **OR** (Surgical Procedure, Colposcopic[Title/Abstract]) **OR** (Surgery, Colposcopic[Title/Abstract]) **OR** (Surgical Procedures, Colposcopic[Title/Abstract]) **OR** (Colposcopic Surgery[Title/Abstract]) **OR** (Colposcopic Surgeries[Title/Abstract]) **OR** (Surgeries, Colposcopic[Title/Abstract])) **AND** ((Diagnosis[MeSH Terms]) **OR** (Diagnoses[Title/Abstract]) **OR** (Diagnose[Title/Abstract]) **OR** (Diagnoses and Examinations[Title/Abstract]) **OR** (Examinations and Diagnoses[Title/Abstract]) **OR** (Diagnoses and Examination[Title/Abstract]) **OR** (Examination and Diagnoses[Title/Abstract]) **OR** (Postmortem Diagnosis[Title/Abstract]) **OR** (Diagnoses, Postmortem[Title/Abstract]) **OR** (Diagnosis, Postmortem[Title/Abstract]) **OR** (Postmortem Diagnoses[Title/Abstract]) **OR** (Antemortem Diagnosis[Title/Abstract]) **OR** (Antemortem Diagnoses[Title/Abstract]) **OR** (Diagnoses, Antemortem[Title/Abstract]) **OR** (Diagnosis, Antemortem[Title/Abstract])) **AND** ((Sensitivity and Specificity[MeSH Terms]) **OR** (Specificity and Sensitivity[Title/Abstract]) **OR** (Sensitivity[Title/Abstract]) **OR** (Specificity[Title/Abstract])) **AND** ((Pathology[MeSH Terms]) **OR** (Pathologies[Title/Abstract]) **OR** (Biopsy[Title/Abstract]) **OR** (Biopsies[Title/Abstract]) **OR** (cytopathology[Title/Abstract]) **OR** (histopathology[Title/Abstract]))

**Web of Science-1490**

#1 TS=(Uterine Cervical Neoplasm) OR TS=(Cervical Neoplasm) OR TS=(Cervical Neoplasm) OR TS=(Cancer of the Uterine Cervix) OR TS=(Cancer of the Cervix) OR

TS=(Cervix Cancer) OR TS=(Squamous Intraepithelial Lesion) OR TS=(High Grade Squamous Intraepithelial Lesion) OR TS=(Low Grade Squamous Intraepithelial Lesion) OR TS=(HSIL) OR TS=(LSIL)

#2 ((((((TS=(Colposcopy)) OR TS=(Colposcopies)) OR TS=(Colposcopic Surgical Procedures)) OR TS=(Colposcopic Surgical Procedure)) OR TS=(Colposcopic Surgical Procedure)) OR TS=(Colposcopic Surgeries)) OR TS=(kolposcopy)

#3 (((((((((TS=(Diagnosis)) OR TS=(Diagnoses)) OR TS=(Diagnose)) OR TS=(Diagnoses and Examinations)) OR TS=(Examinations and Diagnoses)) OR TS=(Postmortem Diagnosis)) OR TS=(Postmortem Diagnoses)) OR TS=(Antemortem Diagnosis)) OR TS=(Antemortem Diagnoses)) OR TS=(Diagnostics)

#4 (((((TS=(Pathology)) OR TS=(Pathobiology)) OR TS=(Pathologies)) OR TS=(Biopsy)) OR TS=(Pathologic institute)) OR TS=(Pathology institute)

#5 (((TS=(Sensitivity and Specificity)) OR TS=(Sensitivity and Specificity)) OR TS=(Sensitivity)) OR TS=(Specificity)

#6 ((((#1) AND #2) AND #3) AND #4) AND #5

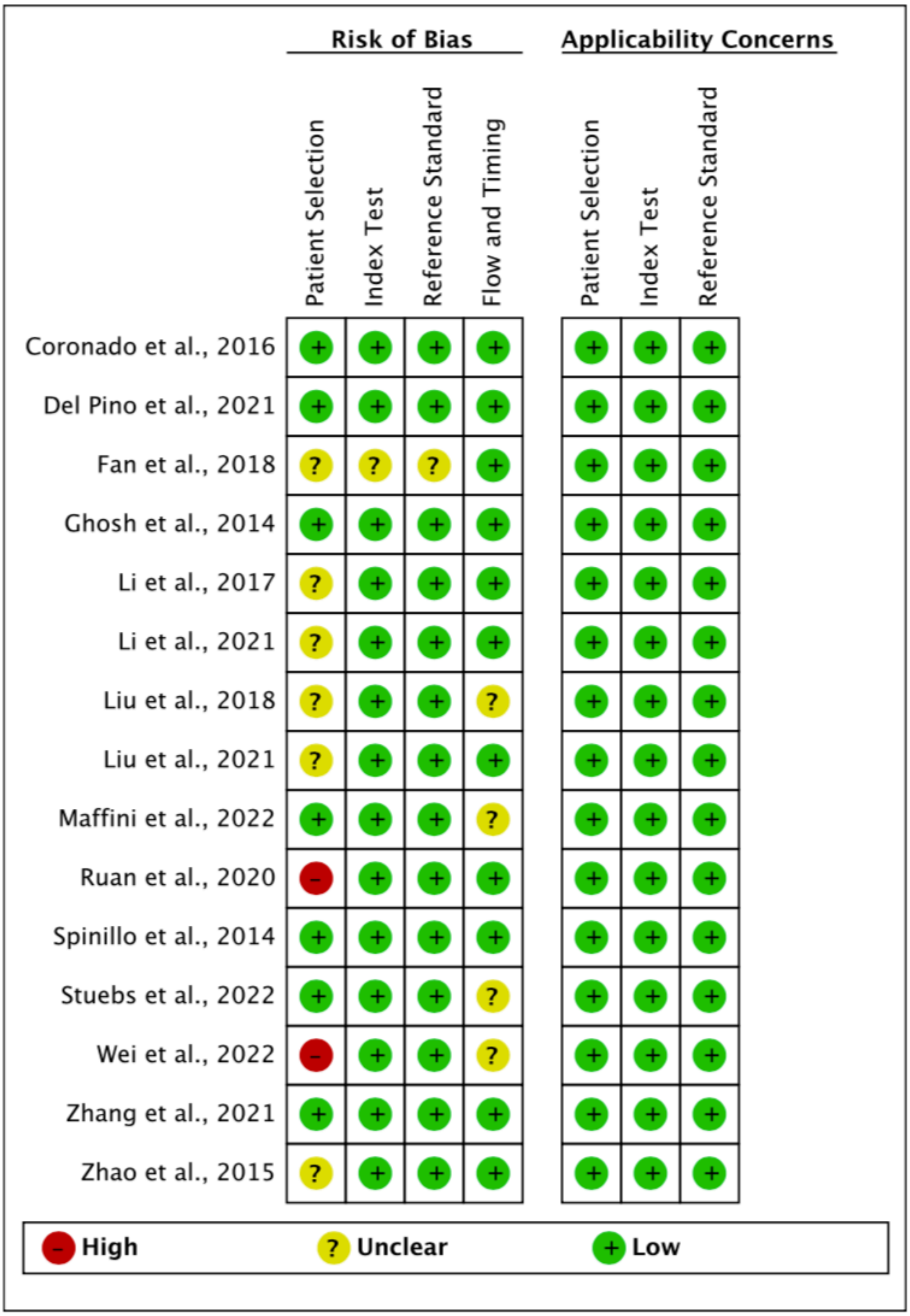


**Figure S1** Details of quality assessment by the QUADAS-2 tool

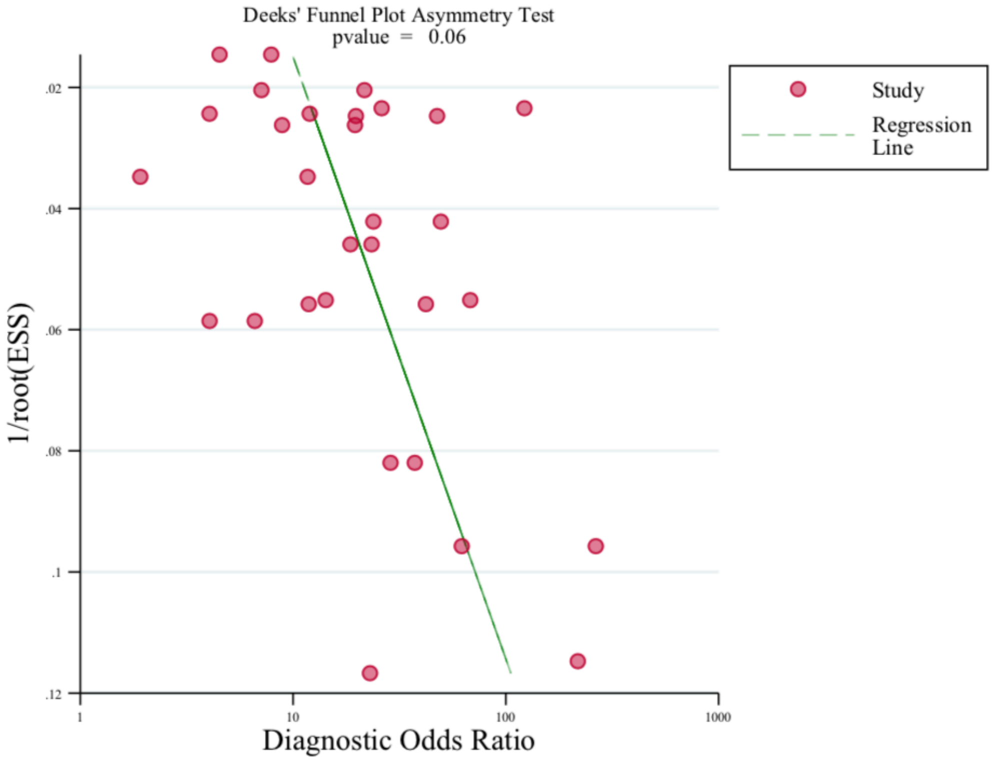


**Figure S2** Deeks’ Funnel Plot Asymmety
